# The Tongue in Disease

**Published:** 1894-12

**Authors:** 


### Selections.

### The Tongue in Disease.

Dr. F. L. Gerald in *Herald of Health* says: The tongue is of great diagnostic value and by close observation it will give us valuable aid in determining the character of disease. The tongue tells us of the condition of the blood, the condition of the nervous system, and the functions of nutrition and excretion. As these are important things to know, in fact just what we want to know, we will make the tongue talk as plainly as possible. We find the expression of disease in its form, its condition of dryness or moisture, its coatings and colors. Change in form is expressive of disease. The elongated and pointed tongue indicates a condition of irritation and determination of blood to the stomach and bowels, and it is safe to give it full weight, and be careful in the administration of remedies.

As it is associated with excitation of the nerve centers, this evidence is valuable with reference to the stomach and bowels. If we observe this change of form at first, we not only anticipate unpleasant gastric irritation during the sickness, but it puts us on our guard against using anything that will irritate the stomach and bowels. The full tongue, broad and thick, is evidence of atony, want of action in the digestive tract. In this case the stomach will bear cathartics in mild form without danger. The dry, pinched tongue expresses a want of functional activity in the digestive organs. It is the tongue of acute disease, and is usually associated with dryness. While it is one of the indications for food, we must be very careful in its selection, giving small quantities at a time, and in a warm liquid form.

The fissured tongue in chronic disease indicates inflammatory action of the kidneys. The fissured tongue in advanced stages of acute disease refers us to lesions of the kidneys, or irritation of the nerve centers. In many cases we find a wrong in the secretion of urine. It deserves close attention and means to put the skin in better condition, and allay irritation of the nerve centers. Dryness and moisture are important evidence of the condition of the digestive organs. If the tongue is dry we are sure the stomach and intestines can do but little digestive work. It is absurd to employ cathartics in such cases unless the object is simply to remove irritating matter. In acute disease with dryness of tongue, when we find it becoming moist, we are confident of improvement, and it is nearly always looked upon as a favorable symptom.

The thin, transparent coating of the tongue gives evidence of enfeebled digestion, frequently from intemperate eating and drinking.

A heavily-loaded tongue at the base calls attention to accumulations in the stomach, and suggests the use of an emetic. The broad, pallid tongue gives evidence of a want of the alkaline elements of the blood. It may be the basis of the entirety of the disease, which will fade away as soon as the proper alkali is given, or it may be but a portion of the wrong, and the alkaline salt prepares the way and facilitates the action of other remedies. The deep red tongue, generally dry, indicates an acid. A dirty white, or dirty gray tongue means antiseptics.

While dryness always indicates excitement of the nerve centers and calls for sedatives, too much moisture and relaxation is evidence of the opposite condition.—*The Medical Brief.*

